# Our Editorial Staff

**Published:** 1871-10

**Authors:** 


### PART III.

### Editorials, Correspondence and Miscellaneous.

### OUR EDITORIAL STAFF.

For ability and learning the Editorial Staff—composed of Associate and Corresponding Editors—of THE COMPANION, cannot be excelled in this country. We again have the pleasure of presenting our readers the names of other gentlemen, well and favorably known to the Profession of the entire Union. Such a "galaxy of names" form "a brotherhood," all must respect and admire. THE COMPANION enters upon a career of power and usefulness in the advocacy of principle, and the up-building of Science, which shall be exerted for the good of the Profession and the interests of humanity.

We call attention to the original department of this number. The communications will be found useful and instructive, sustaining the individual character of our Associate Editors, and others, as writers. We invite all to send us communications. Let THE COMPANION be the means of opening up many a hidden mine of precious mental ore for the glory of Medicine and the honor of Science.

